# Diagnostic Efficacy of Magnifying Endoscopy with Narrow-Band Imaging for Gastric Neoplasms: A Meta-Analysis

**DOI:** 10.1371/journal.pone.0123832

**Published:** 2015-04-09

**Authors:** Xiuhe Lv, Chunhui Wang, Yan Xie, Zhaoping Yan

**Affiliations:** Department of Gastroenterology, West China Hospital of Sichuan University, Chengdu, Sichuan, China; University of Utah Health Sciences Center, UNITED STATES

## Abstract

**Background:**

Magnifying endoscopy with narrow-band imaging (ME-NBI) is a novel, image-enhanced endoscopic technique for differentiating gastrointestinal neoplasms and potentially enabling pathological diagnosis.

**Objectives:**

The aim of this analysis was to assess the diagnostic performance of ME-NBI for gastric neoplasms.

**Methods:**

We performed a systematic search of the PubMed, EMbase, Web of Science, and Cochrane Library databases for relevant studies. Meta-DiSc (version 1.4) and STATA (version 11.0) software were used for the data analysis. Random effects models were used to assess diagnostic efficacy. Heterogeneity was tested by the Q statistic and *I^2^* statistic. Meta-regression was used to analyze the sources of heterogeneity.

**Results:**

A total of 10 studies, with 2151 lesions, were included. The pooled characteristics of these studies were as follows: sensitivity 0.85 (95% confidence interval [CI]: 0.81–0.89), specificity 0.96 (95% confidence interval [CI]: 0.95–0.97), and area under the curve (AUC) 0.9647. In the subgroup analysis, which compared the diagnostic efficacy of ME-NBI and white light imaging (WLI), the pooled sensitivity and specificity of ME-NBI were 0.87 (95% CI: 0.80–0.92) and 0.93 (95% CI: 0.90–0.95), respectively, and the area under the curve (AUC) was 0.9556. In contrast, the pooled sensitivity and specificity of WLI were 0.61 (95% CI: 0.53–0.69) and 0.65 (95% CI: 0.60–0.69), respectively, and the area under the curve (AUC) was 0.6772.

**Conclusions:**

ME-NBI presents a high diagnostic value for gastric neoplasms and has a high specificity.

## Introduction

Gastric neoplasms, which constitute the third leading cause of death due to cancer, continue to be an important health threat worldwide and cause both medical and economic burdens globally [1]. Endoscopy is currently a major method for gastric cancer screening because of its high detection rate, but its diagnostic accuracy depends heavily on the availability of endoscopic instruments [2].Several novel image-enhancement endoscopic techniques have been used in recent years for the detection of gastrointestinal neoplasms, such as magnifying endoscopy, narrow-band imaging, autofluorescence imaging, and endoscopic microscopy. Magnifying endoscopy with narrow-band imaging (ME-NBI), a combination of magnification endoscopy and narrow-band imaging, has been used specifically for the detection of early gastric cancer. Its success is based on its ability to yield clear images of microvascular patterns and microsurface structures of the superficial mucosa, which represent neoplasia-specific abnormalities. Several recent studies have described correlations between ME-NBI appearance and pathology of both non-neoplastic and neoplastic lesions [3–7]. Several studies have also attempted to assess the diagnostic performance of ME-NBI compared with pathological findings or other image-enhancement endoscopic techniques. To determine the utility of ME-NBI as a novel endoscopic technique, in this study, we performed a meta-analysis to assess the diagnostic efficacy of magnifying endoscopy with narrow-band imaging for gastric neoplasms.

## Materials and Methods

### Search strategy

A systematic search was performed of the PubMed, EMbase, Cochrane Library, and Web of Science databases for relevant published studies up to September 28, 2014. The following search terms were used: “magnifying endoscopy,” “narrow-band imaging,” “ME-NBI,” “M-NBI,” “gastric neoplasm,” and “gastric cancer.” The references in the available articles were also reviewed. Two investigators independently searched the references for applicable studies, and disagreements were resolved by discussion.

### Inclusion and exclusion criteria

Studies were selected according to the following inclusion criteria: 1) diagnostic clinical trials that used ME-NBI to differentiate gastric neoplastic lesions from non-neoplastic lesions; 2) pathology from biopsy or endoscopic or surgical treatment was used as the reference standard (gold standard) for the diagnosis of lesions; 3) sensitivity and specificity were reported or could be calculated from 2×2 contingency tables; 4) absolute numbers of true-positive (TP), false-positive (FP), true-negative (TN), and false-negative (FN) cases were provided; and 5) full-text articles could be obtained in English. Studies with the following characteristics were excluded: 1) patients without gastric lesions but with other lesions, such as esophageal and colonic lesions, or patients who were reported in duplicate in similar studies; 2) data without pathological confirmation of lesions or that could not be fully extracted from the published information; and 3) unsuitable publication types, including comments, reviews, guidelines, or case reports. We did not attempt to contact the authors.

### Data extraction

The following data were collected: the study characteristics, including first author’s name, year of publication, country of origin, study design, reference standard, endoscopic classification, number of endoscopists, and type of endoscopes, and the patient characteristics, including numbers of patients and lesions, age, and gender. Sensitivity and specificity were recorded from the studies, and the numbers of TP, FP, TN, and FN were extracted for the construction of 2×2 tables. Two investigators independently extracted the data and crosschecked the results.

### Qualitative assessment

The included articles were assessed by the Quality Assessment of Diagnostic Studies-2 (QUADAS-2). This tool is designed specifically for evaluating the risk of bias and applicability of primary diagnostic accuracy studies, and it is focused on the 4 following key domains that are rated in terms of the risk of bias: patient selection, index test, reference standard, and flow and timing [8]. According to the answers to signaling questions (Yes/No/Unclear) and to the judgment of concerns regarding applicability in relevant domains, the bias or applicability of a study is judged as “low,” “high,” or “unclear.” If the study is judged as “low” in all domains related to bias or applicability, the overall judgment should be “low risk of bias” or “low concern regarding applicability.” In contrast, if the judgment is “high” in one or more domains, the relevant study should be judged as having a “high risk of bias” or “high concerns regarding applicability.” “Unclear” is used in cases in which insufficient data are obtained, which can cause difficulties in judgment. The quality assessment was also performed and crosschecked independently by two reviewers.

### Statistical analysis

For the included studies, the possibility of a threshold effect was first tested by calculating the Spearman correlation coefficient. We pooled the sensitivity, specificity, likelihood ratio, and diagnostic odds ratio (DOR) by using a random effects model to derive estimates and 95% confidence intervals (CIs). This was combined with the creation of a symmetric receiver operator characteristic (SROC) curve if the evidence of a threshold effect was not found. The area under the SROC curve (AUC) and integrated DOR value, which was computed using a random effects model, were used to analyze the diagnostic efficacy of ME-NBI if the evidence of a threshold effect was found. The heterogeneity of the studies was assessed using the Q statistic and *I* ^*2*^ statistic, and *I* ^*2*^ values of 25%, 50%, and 75% were considered to represent low, moderate, and high inconsistency, respectively [9]. Meta-regression was used to identify the sources of heterogeneity.

We also performed a subgroup analysis to evaluate the diagnostic efficacy of ME-NBI compared with that of conventional white light imaging (WLI). Because other diagnostic modalities may exist and confound the results, we qualified the studies and selected those with independent efficacy descriptions for both ME-NBI and WLI, with the exclusion of other modalities.

All data analyses in this study were performed using Meta-DiSc (version 1.4) and STATA (version 11.0, College Station, TX) software. The Meta-DiSc software was used to test the threshold effect between studies and to combine the indexes used to describe the diagnostic efficacy of ME-NBI. The STATA software was used for meta-regression analysis.

## Results

### Search results

**Fig 1:**
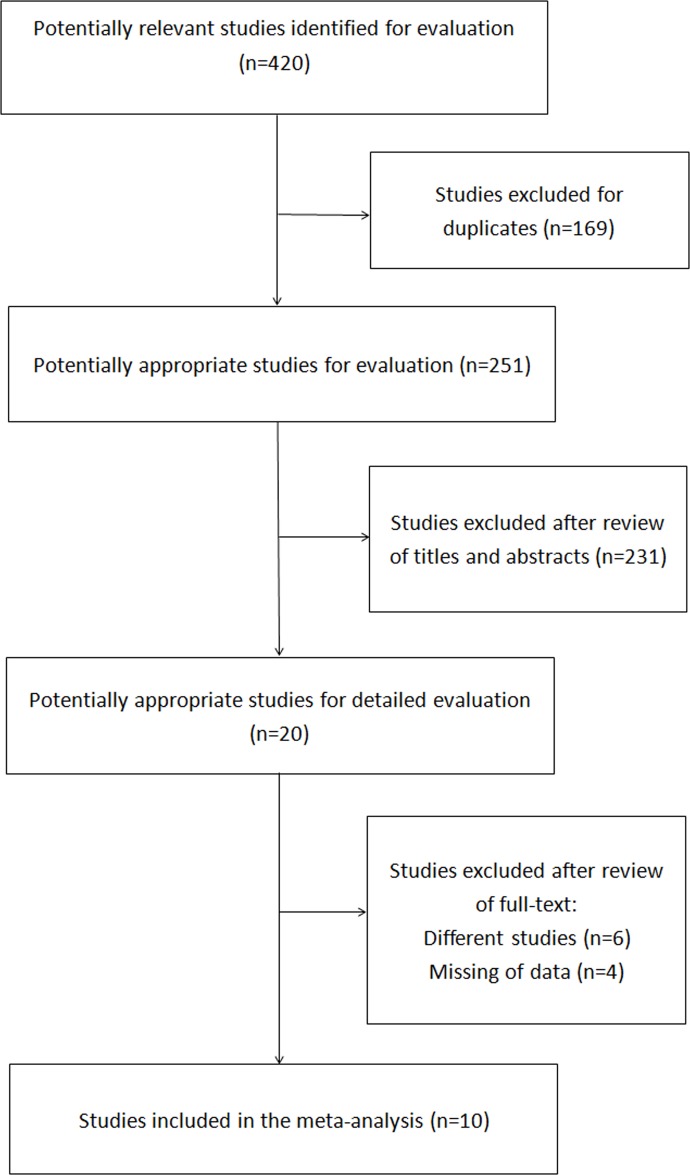
Flow diagram showing the selection process of articles.

The initial search identified 420 articles. Of these articles, 20 relevant studies were selected for detailed evaluation after the exclusion of duplicates and a review of the remaining titles and abstracts. Finally, 10 studies were included in our analysis (Fig 1). The reasons for the final exclusion of studies included the following: 1) different evaluation methods for diagnostic efficacy were used [7,10,11]; 2) the main outcome was defined as invasion depth [12,13] and tumor margin [14]; and 3) sufficient data were not provided to enable calculation of the sensitivity and specificity or construction of the 2×2 table [15–18]. The features of the included studies are summarized in Table 1.

**Table 1:** Characteristics of the studies selected for the meta-analysis.

Author (year)	Country	Number of lesions (n)	Number of patients (n)	Mean age (years)	M/F	Endoscopy type	Diagnostic classification	Pathological reference standard	Number of endoscopists	Study design	Limitations of the analysis
Ezoe Y, 2010[19]	Japan	57	53	NS	NS	GIF-Q240Z/GIF-H260Z	Yao et al	Revised Vienna classification	5	Prospective	Small depressive lesions (≤10 mm)
Kato M, 2010[20]	Japan	201	111	66.3	98/13	GIF-H260Z	Kaise et al	Revised Vienna classification	NS	Prospective	None
Ezoe Y, 2011[21]	Japan	177	177	69	140/37	GIF-Q240Z/ GIF-H260Z/GIF-FQ260Z	Yao et al	Revised Vienna classification	31	Prospective	Small depressive lesions (≤10 mm)
Li H. Y, 2012[22]	China	164	146	59.3	88/58	GIF-H260Z	Original	Revised Vienna classification +WHO classification	2	Prospective	None
Miwa K, 2012[25]	Japan	135	135	70.1	77/58	GIF-Q240Z/ GIF-H260Z	Yao et al	NS	7	Retrospective	Adenomas
Maki S, 2013[26]	Japan	93	NS	NS	NS	GIF-Q240Z/ GIF-H260Z	Yao et al	Revised Vienna classification	2	Retrospective	Elevated gastric lesions
Fujiwara S, 2014[27]	Japan	103	99	NS	69/30	GIF-Q240Z/GIF-H260Z	Yao et al	Revised Vienna classification	2	Retrospective	Minute gastric lesions (≤5 mm)
Guo T, 2014[28]	China	643	508	63	316/192	GIF-H260Z	Yao et al	Revised Vienna classification	4	Retrospective	None
Liu H, 2014[23]	China	207	90	57.5	49/41	GIF-H260Z	Original	WHO classification	2	Prospective	Antral Lesions
Yao K, 2014[24]	Japan	371	310	66	183/127	GIF-Q240Z/ GIF-H260Z	Yao et al	Revised Vienna classification	20	Prospective	None

NS, not stated; Yao et al, the classification system proposed by Yao et al. [29]; Kaise et al, the classification system proposed by Kaise et al. [30]; Original, the classification system proposed by the authors themselves; None, the study was conducted without following specific requirements.

### Quality assessment

The quality assessment of the eligible studies is shown in Table 2. Generally, the included studies met most of the quality criteria. A major problem we found was that the use of a blinding method for a reference standard was not implemented or mentioned in six studies, which may have resulted in an unknown risk or high risk of bias in relevant articles. Furthermore, one study used a case-control design, resulting in both high risks of bias and high concerns regarding applicability.

**Table 2:** Quality assessment of the studies selected for the meta-analysis (QUADAS-2).

Study	Risk of Bias				Applicability Concerns		
	Patient Selection	Index Test	Reference Standard	Flow and Timing	Patient Selection	Index Test	Reference Standard
**Ezoe Y. 2010**	L	L	U	L	L	L	L
**Kato M, 2010**	L	L	H	L	L	L	L
**Ezoe Y, 2011**	L	L	U	L	L	L	L
**Li H. Y, 2012**	L	L	L	L	L	L	L
**Miwa K, 2012**	L	L	U	L	L	L	L
**Maki S, 2013**	L	L	U	L	L	L	L
**Fujiwara S, 2014**	H	L	L	L	H	L	L
**Guo T, 2014**	L	L	H	L	L	L	L
**Liu H, 2014**	L	L	L	L	L	L	L
**Yao K, 2014**	L	L	L	L	L	L	L

L, low risk; H, high risk; U, unclear risk.

### Analysis results

**Fig 2:**
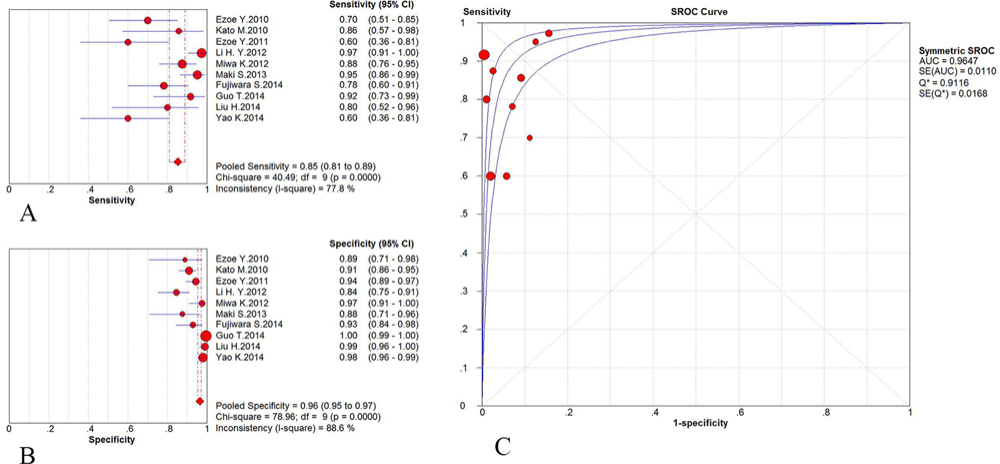
Analysis of the diagnostic efficacy results of ME-NBI. A: Pooled sensitivity of ME-NBI for diagnosing gastric neoplasms. B: Pooled specificity of ME-NBI for diagnosing gastric neoplasms. C: Symmetric receiver operating characteristic (SROC) curve and area under the curve (AUC).

Six prospective studies [19–24] and four retrospective studies [25–28] were included, comprising a total of 2151 lesions. For these studies, the Spearman correlation coefficient was 0.322 (*p* = 0.364), which indicated that the lack of a definite threshold effect induced heterogeneity. The Cochran’s Q and *I* ^*2*^ for the DOR were 28.19 (*p* = 0.0009) and 68.1%, respectively, which indicated a moderate level of heterogeneity induced by the non-threshold effect. When a random effects model was used, the pooled characteristics were as follows: sensitivity 0.85 (95% CI: 0.81–0.89), specificity 0.96 (95% CI: 0.95–0.97), positive likelihood ratio 16.70 (95% CI: 9.21–30.31), negative likelihood ratio 0.18 (95% CI: 0.11–0.30), and diagnostic OR 106.25 (95% CI: 47.03–240.01). The AUC was 0.9647 (SE = 0.0110) with Q* = 0.9116 (SE = 0.0168), indicating a high level of diagnostic accuracy for ME-NBI in gastric neoplasms (Fig 2).

**Fig 3:**
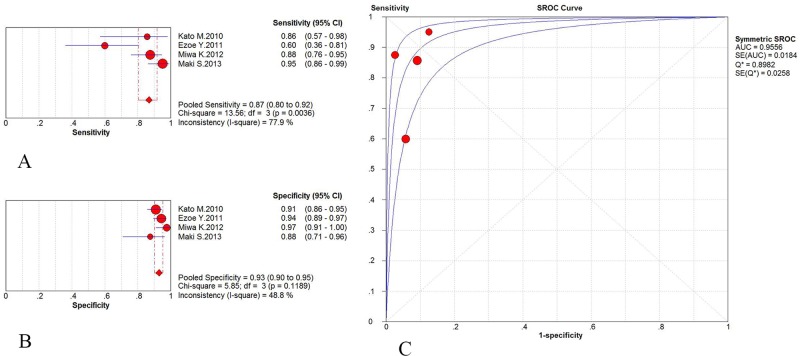
ME-NBI results from the subgroup analysis performed to evaluate the diagnostic efficacy of ME-NBI vs. WLI. A: Pooled sensitivity of ME-NBI in the subgroup analysis. B: Pooled specificity of ME-NBI in the subgroup analysis. C: Symmetric receiver operating characteristic (SROC) curve and area under the curve (AUC).

**Fig 4:**
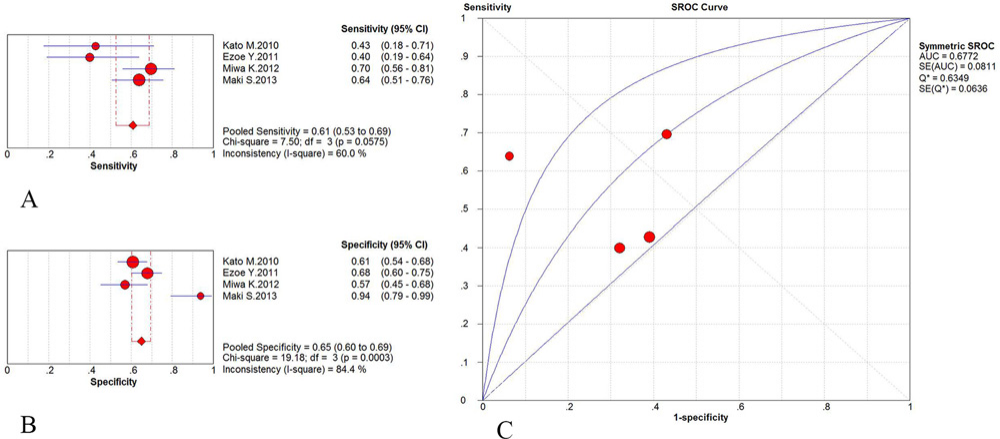
WLI results from the subgroup analysis performed to evaluate the diagnostic efficacy of ME-NBI vs. WLI. A: Pooled sensitivity of WLI in the subgroup analysis. B: Pooled specificity of WLI in the subgroup analysis. C: Symmetric receiver operating characteristic (SROC) curve and area under the curve (AUC).

The subgroup analysis performed to evaluate the diagnostic efficacy of ME-NBI and WLI was carried out using data from four articles. All of the articles were from Japan, and they included two prospective studies [20,21] and two retrospective studies [25,26]. According to the Spearman correlation coefficient and *I* ^*2*^ for the DOR, as described previously, there was no definite threshold effect-induced heterogeneity found for these studies. The pooled sensitivity and specificity for ME-NBI were 0.87 (95% CI: 0.80–0.92) and 0.93 (95% CI: 0.90–0.95), respectively, and the AUC was 0.9556 (Fig 3). The pooled sensitivity and specificity for WLI were 0.61 (95% CI: 0.53–0.69) and 0.65 (95% CI: 0.60–0.69), respectively, and the AUC was 0.6772 (Fig 4).

### Heterogeneity test

To explore possible sources of the heterogeneity, we performed a meta-regression analysis. The specific variables assessed included the following: study design (prospective or retrospective), diagnostic classification (proposed by Yao et al. or not), pathological reference standard (revised Vienna classification used or not), and restriction setting (present or absent). However, as shown in Table 3, none of the factors had any definite influence on heterogeneity. Heterogeneity may have arisen due to other reasons, such as the number of included patients, age and sex ratio, differences in the operating protocol, and the use of a blinding method, which could not be analyzed in the present study due to the partial loss of data or unrecognizable details.

**Table 3:** Results of the meta-regression performed to identify potential sources of heterogeneity.

Specific variables	Coefficient	*P* value	Relative DOR (95% CI)
Study design	-1.821	0.1177	0.16 (0.01–2.06)
Diagnostic classification	-0.750	0.6079	0.47 (0.01–20.01)
Reference standard	-1.472	0.3231	0.23 (0.01–8.64)
Restriction setting	-1.615	0.1442	0.20 (0.02–2.36)

CI, confidence interval.

## Discussion

It is widely known that the highest incidence rates of gastric neoplasm occur in East Asia. This “Asian type” of cancer is associated with poverty and is strongly correlated with *H*. *pylori* infection to a greater degree than other types of cancers [31]. The formation of gastric cancer is a multistep process that includes the sequential development of chronic gastritis, mucosal atrophy, intestinal metaplasia, adenoma, and early gastric cancer [32]. These early stages of carcinogenesis cannot be detected, which usually results in a high mortality rate for gastric cancer. As a novel observational method, ME-NBI has been used in recent years to solve this problem, and it has been shown to facilitate the detection of changes associated with metaplasia, dysplasia, or cancer [36]. It has also facilitated pathological diagnosis through observation, without the need to perform a biopsy [33]. Compared with pathological findings, our meta-analysis suggests that ME-NBI can achieve a high level of diagnostic accuracy for gastric neoplasms.

Many image-enhanced endoscopic techniques have been shown to increase the diagnostic yield for characterization of early gastric cancer [34]. However, there are limitations for using such endoscopic techniques solely to detect subtle changes of the gastric mucosa. Magnifying endoscopy enables image magnification compared with traditional endoscopy, and high-magnification endoscopy (HME) can enlarge an image up to 100-fold [35]. Nevertheless, conventional employment of magnification endoscopy in the stomach can be difficult, mainly because the optical resolution of endoscopes is insufficient to facilitate clear visualization of the mucosal surface and blood vessels [24]. The narrow-band imaging (NBI) system is an optical technique used for enhanced visualization of microvascular architecture and microsurface structures using narrower bands of blue and green filters. However, the weak light intensity of NBI usually leads to dim, low contrast images, which significantly limits its utility [36]. Based on the strengths and weaknesses of these two image-enhancement endoscopic technologies, ME-NBI is expected to exhibit the greatest utility in accurately diagnosing gastric cancer by enabling a clear visualization of microvascular patterns and microsurface structures of the superficial mucosa. Many diagnostic classification systems have been introduced to describe ME-NBI findings. Although the distinctions exist between these classification systems [5, 29, 30, 37], the ME-NBI findings of irregular microvascular pattern with a demarcation line and/or irregular microsurface pattern with a demarcation line have been proposed to be crucial for accurate endoscopic diagnosis of gastric neoplasms [24, 38]. On the other hand, autofluorescence (AFI) endoscopy and microscopic endoscopy have been considered to be feasible endoscopic techniques for the detection of early gastric cancer as well. However, the role of AFI in detecting gastric neoplasia is limited by its poor specificity, and the specificity of AFI is actually lower than that of WLI because of higher false positives [39]. The appliance of microscopic endoscopy still requires the intravenous administration or direct spraying of fluorescent dye, and the skills of this technology can be difficult to grasp [36]. These problems can be solved better with ME-NBI in consideration of its high specificity and convenience. Furthermore, white light imaging endoscopy (WLI) has been widely used in clinical practice, but it has difficulty differentiating between non-malignant and early neoplastic lesions. In this study, the ME-NBI group obtained higher sensitivity and specificity than the WLI group, leading to our recommendation to assist WLI conventionally for the inspection of lesions in the near future.

Notably, ME-NBI plays important roles in the diagnosis of gastric neoplasms in ways other than the capability to differentiate cancerous and noncancerous lesions. For example, it may be useful for predicting the depth of invasion. Kobara et al. [12] proposed a scoring system based on three indicators, namely, “non-structure,” “scattery vessel,” and “multi-caliber vessel,” and the presence of two or more indicators found by ME-NBI was considered to be highly correlated with the SM2 invasion depth of depressed-type gastric cancer. Kikuchi et al. [13] provided a much simpler indicator, namely “dilated vessels” (D vessels), with a high diagnostic accuracy of 81.5% for assessing the depth of invasion in different gastric cancers. Furthermore, ME-NBI has also been used to determine the gastric tumor margin. Kiyotoki et al. [14] compared the ability of ME-NBI and indigo carmine chromoendoscopy (ICC) to detect tumor margins and showed that the tumor margins could be identified more clearly by ME-NBI than by ICC (97.4% vs. 77.8%; p = 0.009). These results suggest that ME-NBI has multiple uses in the diagnosis of gastric neoplasms, and these uses will be valuable for the endoscopists to decide the location and size of lesions for taking biopsies as well. ME-NBI has demonstrated high specificity in our study, and this could be particularly useful for reducing unnecessary biopsies during the endoscopic inspection process because patients with non-malignant lesions would be excluded from such procedures.

The treatment of early gastric cancer often requires therapeutic endoscopies. Therapeutic endoscopic techniques, which usually refer to endoscopic mucosal resection (EMR) and endoscopic submucosal dissection (ESD), are commonly chosen based on the pathological stage at the time of detection, and sufficient biopsy specimens are needed to determine the pathological stage of lesions. Our study has demonstrated the high diagnostic efficacy of ME-NBI, which aims to provide better visualization to differentiate cancerous lesions and to validate pathological diagnoses. This would be advantageous for endoscopists to determine pathological stages of lesions and to clarify the decision to use therapeutic endoscopic techniques.

There are several limitations of our study. First, we only included 10 studies, and some of them were not of high quality. A blinding method for the interpretation of pathology results was either not used or not mentioned in 6 studies. More multicenter studies with strict controls on experimental procedures are needed to provide high-quality data for further analysis. Second, the pooled sensitivity and specificity in this study were based on per-lesion analysis. Although the numbers of patients were provided in 9 studies except one [26], none of these articles provided specific patient characteristic data. Thus, there may be some differences in the results obtained from the per-patient analysis when compared with the per-lesion analysis. Third, heterogeneity existed between different studies. Because the meta-regression did not identify the sources of heterogeneity, we could only speculate on the potential reasons for the heterogeneity. Thus, more high-quality data are needed to address this problem. Fourth, in the subgroup analysis, we only compared the diagnostic accuracy of ME-NBI and WLI; the comparisons of ME-NBI with other image-enhanced endoscopic techniques were not included, mainly due to the lack of sufficient data. More controlled trials are needed to explore different application values of these endoscopic techniques. Finally, we only included studies published in English from four major databases. However, a systematic search for relevant studies in Chinese databases was also conducted, and no useful reports were found. Nevertheless, some important articles from other countries may have been missed in this review.

## Conclusions

In conclusion, this meta-analysis demonstrates the high diagnostic value and high specificity of ME-NBI for gastric cancers. More prospective studies with high-quality designs should be performed to further validate these findings.

## Supporting Information

S1 PRISMA Checklist (DOC)
